# Minimal residual disease and relapse surveillance in osteosarcoma: an action-linked framework integrating liquid biopsy and imaging biomarkers

**DOI:** 10.1016/j.jbo.2026.100803

**Published:** 2026-09-16

**Authors:** Guoli Huang, Yang Zhou, Hao Wang, Xiucai Ma

**Affiliations:** a First Clinical Medical School of Gansu University of Chinese Medicine, Lanzhou, PR China.; b Department of Bone and Soft Tissue Oncology, Gansu Provincial People's Hospital, Lanzhou, Gansu, PR China.

**Keywords:** Osteosarcoma, Minimal residual disease, Relapse surveillance, Liquid biopsy, Circulating tumor DNA, Imaging biomarkers, Radiomics

## Abstract

Osteosarcoma relapse surveillance remains dominated by scheduled imaging because salvage treatment depends on anatomical confirmation of pulmonary, local or extrapulmonary recurrence. However, radiological recurrence may occur after a biologically active phase in which residual viable disease or micrometastatic progression is already present but not yet localizable. This clinical-translational review reframes postoperative osteosarcoma surveillance as an action-linked decision workflow rather than a comparison of isolated biomarker technologies. Current evidence suggests that tumor-informed circulating tumor DNA (ctDNA) sequencing provides the strongest osteosarcoma-specific minimal residual disease (MRD) signal, with postoperative positivity associated with inferior event-free survival and, in selected patients, molecular detection preceding imaging-confirmed relapse or progression. Cell-free DNA methylation may offer a mutation-independent adjunct, whereas circulating tumor cells, extracellular vesicles and circulating microRNAs remain exploratory signals without validated postoperative surveillance actions. Chest computed tomography (CT) and local magnetic resonance imaging (MRI) remain indispensable for disease localization and treatment planning, while diffusion-weighted imaging, dynamic contrast-enhanced MRI and radiomics currently provide mainly local viability or risk-enrichment information rather than proven surveillance-intervention evidence. The near-term role of integrated biomarkers is therefore not to replace guideline-based imaging, but to define protocolized pathways for molecular-positive/imaging-negative, imaging-positive/molecular-negative, concordant high-risk and concordant low-risk states. Future studies should test whether biomarker-triggered reassessment improves clinically meaningful outcomes, including resectability, second complete remission, clinical trial access, patient burden and survival, rather than simply documenting recurrence earlier.

## Highlights

• Osteosarcoma surveillance should link biomarker signals to clinical actions.
• Tumor-informed circulating tumor DNA (ctDNA) best supports residual disease detection.
• Chest computed tomography (CT) and local magnetic resonance imaging (MRI) localize relapse.
• Methylation, circulating cells, vesicles and microRNAs remain exploratory.
• Future trials must test resectability, burden, trial access and survival.

## Graphical abstract

**Figure:**
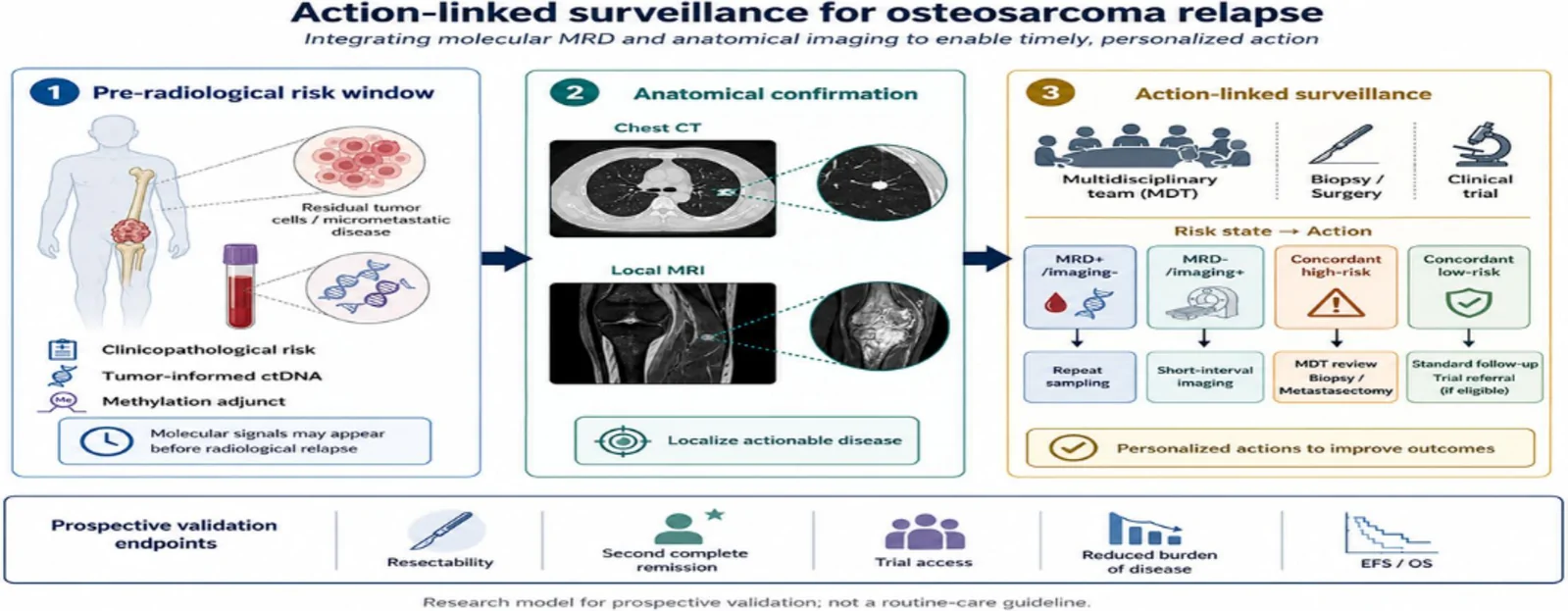
Action-linked surveillance workflow for osteosarcoma relapse.

## 1 Introduction

### 1.1 Relapse as the decisive surveillance problem in osteosarcoma

Modern osteosarcoma treatment is already intensive and multidisciplinary. For resectable high-grade disease, standard management combines preoperative and postoperative multi-agent chemotherapy, most commonly high-dose methotrexate, doxorubicin and cisplatin (MAP), with complete resection of all detectable disease [1], [2]. This strategy transformed outcomes compared with surgery alone, but therapeutic gains have slowed. In the European and American Osteosarcoma Study (EURAMOS-1) cohort, which treated more than 2000 patients within a harmonized international framework, 5-year event-free survival and overall survival were 54% and 71%, respectively, despite standardized MAP-based therapy and expert multidisciplinary care [3]. Earlier cooperative-group experience likewise showed long-term event-free survival stabilizing near 50% for high-grade extremity or trunk osteosarcoma [4], and postoperative intensification with ifosfamide and etoposide for poor histological responders failed to improve event-free survival while increasing toxicity in EURAMOS-1 [5].

The surveillance problem follows directly from this therapeutic plateau. Approximately 30%–40% of patients develop recurrent disease, most often in the lungs, followed by bone metastases and local recurrence [2], [6], [7], [8]. Recurrent osteosarcoma is clinically heterogeneous: late, solitary, unilateral pulmonary recurrence may remain amenable to complete metastasectomy, whereas early relapse, multiple nodules, bilateral lung disease, pleural disruption, extrapulmonary metastases, local recurrence with metastasis, or failure to achieve a second complete remission define less favorable states [6], [7], [9], [10], [11], [12], [13], [14]. Across recurrent-disease series and systemic-therapy reviews, 5-year survival for unselected relapsed patients commonly remains approximately 20%–30% [7], [8], [10]. Even favorable relapse is not uniformly curable; contemporary Cooperative Osteosarcoma Study Group (COSS) data on solitary pulmonary relapse reported approximately 51% 5-year post-relapse overall survival but only 31% post-relapse event-free survival [6]. Surveillance should therefore be judged by whether it preserves a treatable local or metastatic state, not merely by whether recurrence is detected earlier on a calendar.

### 1.2 The biological-radiological gap as a translational target

Post-treatment follow-up remains imaging-centered because most actionable relapses are anatomical events that require radiological confirmation before metastasectomy, biopsy, radiotherapy or surgical planning [2], [15], [16], [17], [18]. Radiological visibility, however, is unlikely to mark the biological beginning of recurrence. Residual viable cells, micrometastatic expansion and molecular relapse may precede a measurable pulmonary nodule or local mass. In other solid tumors, circulating tumor DNA (ctDNA)-based minimal residual disease (MRD) monitoring is being developed to detect this pre-radiological window, although clinical use requires evidence that molecular lead time can be converted into better management or outcomes [19], [20]. Osteosarcoma makes this task difficult because structural variants, copy-number alterations and other complex genomic changes often provide more informative ctDNA targets than recurrent hotspot mutations [21], [22], [23], [24]. Nevertheless, recent osteosarcoma-specific data show that tumor-informed ctDNA surveillance can stratify postoperative relapse risk and may precede imaging-confirmed relapse in selected patients [25].

This biological-radiological gap is clinically meaningful only if it can be converted into an actionable interval. A molecular signal that appears before radiological relapse may create an opportunity for earlier anatomical reassessment, multidisciplinary team (MDT) review, metastasectomy evaluation, trial referral or local therapy planning; however, it may also introduce lead-time bias, false-positive investigations and surveillance-related anxiety if no predefined response is available [19], [20], [26]. Conversely, an imaging abnormality remains actionable even when a molecular assay is negative, because ctDNA shedding may be low, anatomically confined recurrence may release little tumor-derived DNA into plasma, and patient-specific assays may fail to capture emerging subclones. The translational question is therefore not whether liquid biopsy or imaging can detect more signals, but whether those signals can be linked to decisions that improve resectability, second complete remission, trial access, patient burden or survival.

### 1.3 Surveillance as a clinical decision problem, not a detection problem

Detection evidence and decision evidence are not the same. Liquid biopsy may provide molecular timing, but it does not localize disease for biopsy, metastasectomy or radiotherapy. Chest computed tomography (CT) and local magnetic resonance imaging (MRI) provide anatomical localization, but they usually become positive only after disease reaches a visible structural threshold. Functional MRI and radiomics may enrich risk by capturing local viability, perfusion or imaging heterogeneity, but most available evidence still derives from response assessment, prognosis or retrospective model development rather than prospective surveillance intervention. Biomarker-guided follow-up therefore requires a predefined action map in which molecular assays, anatomical imaging, quantitative imaging and MDT interpretation have complementary roles rather than competing claims.

Against this background, this review reframes osteosarcoma relapse surveillance as a decision-linked workflow rather than a technology-comparison exercise. While recent clinical guidelines and expert recommendations have addressed imaging-based follow-up and emerging liquid biopsy approaches in sarcoma, they have not yet established how these signals should be operationally linked to surveillance decisions [2], [20], [27], [28]. We therefore focus on postoperative and follow-up settings and organize evidence by surveillance function—molecular timing, anatomical localization, imaging-derived risk enrichment, discordant-result management and clinical utility—aiming to define how liquid biopsy and imaging biomarkers can be tested within structured pathways rather than to replace guideline-based imaging.

## 2 Methods

This structured clinical-translational narrative review synthesizes evidence on MRD assessment and relapse surveillance in osteosarcoma, with emphasis on liquid biopsy and imaging biomarkers that may complement current follow-up. PubMed/MEDLINE, Embase and Web of Science were searched from database inception to February 2026, supplemented by manual screening of relevant guidelines, systematic reviews and original studies. Search concepts combined osteosarcoma with relapse, recurrence, surveillance, follow-up, minimal residual disease, liquid biopsy, circulating tumor DNA, cell-free DNA methylation, circulating tumor cells, extracellular vesicles, microRNA, CT, MRI, diffusion-weighted imaging, apparent diffusion coefficient, dynamic contrast-enhanced MRI, radiomics and radiogenomics.

Priority was given to osteosarcoma-specific clinical, translational and imaging studies reporting postoperative sampling, biomarker dynamics, radiological progression, relapse, event-free survival, overall survival, treatment response, metastatic risk, resectability or molecular/imaging lead time. Broader pediatric solid-tumor, sarcoma and solid-tumor MRD literature was included selectively when osteosarcoma-specific evidence was limited or when it informed assay validity, imaging methodology, clinical utility or implementation standards. Animal-only and purely mechanistic studies were used only for biological context and were not treated as direct surveillance-implementation evidence.

Because the available literature was highly heterogeneous with respect to biomarker platforms, imaging protocols, sampling schedules, surveillance intervals and outcome definitions, evidence was synthesized qualitatively rather than quantitatively. Accordingly, no formal risk-of-bias assessment or meta-analysis was undertaken.

## 3 Current surveillance and the biological-radiological gap

### 3.1 Imaging-centered follow-up in current practice

Current follow-up strategies are organized around scheduled clinical review and imaging. The European Society for Medical Oncology (ESMO)-EURACAN-GENTURIS-ERN PaedCan guideline, National Comprehensive Cancer Network (NCCN) Bone Cancer update, National Cancer Institute (NCI) Physician Data Query (PDQ) evidence summary, updated British Sarcoma Group guideline and Children's Oncology Group/Society for Pediatric Radiology (COG/SPR) imaging white paper all frame follow-up around detection of pulmonary metastasis, local recurrence, treatment-related morbidity and survivorship needs at a stage where intervention remains feasible [2], [15], [16], [17], [18]. In practice, follow-up includes physical examination, assessment of functional and reconstructive complications, imaging of the primary site with radiography, MRI or CT, and chest surveillance with radiography or CT [2], [17], [18]. Schedules are front-loaded because relapse risk is highest during the early post-treatment period; ESMO suggests visits approximately every 2–3 months during the first 2 years, every 6 months in years 3–5, every 6–12 months in years 5–10, and then individualized follow-up according to local practice and patient factors [2].

### 3.2 Chest CT, chest radiography and the problem of actionable detection

The unresolved issue is not whether imaging is needed, but how intensive, how long and how adaptive it should be. Osteosarcoma-specific follow-up series support early, lung-focused surveillance but do not establish a universally optimal imaging modality or interval [29], [30]. The practical interpretation of the CT-versus-radiography literature is not that one modality should replace all others, but that earlier radiological detection is valuable only when it preserves a salvage option. Paioli et al. reported that CT-based follow-up was associated with higher second complete remission rates and better post-relapse outcomes than chest radiography in selected patients with lung-only recurrence, whereas Rothermundt et al. argued from a smaller extremity osteosarcoma cohort that chest radiography could detect lung relapse when metastasectomy remained possible and that routine CT should be balanced against radiation exposure [29], [30]. The updated randomized TOSS study across limb sarcomas similarly found no survival disadvantage with less intensive surveillance despite earlier pulmonary metastasis detection by CT [31]. Thus, current evidence supports imaging as the anatomical backbone while also showing why surveillance innovation must be judged by actionable outcomes, not by earlier detection alone.

### 3.3 From molecular lead time to actionable lead time

The biological-radiological gap is the interval between disease activity at a molecular or microscopic level and recurrence that can be confidently localized on CT or MRI. For surveillance design, this gap is not merely a biological concept; it is a decision interval in which clinicians must decide whether a non-anatomical signal is sufficiently credible to justify earlier imaging or MDT review. Osteosarcoma relapse is usually recorded when a pulmonary nodule, local mass or metastatic lesion becomes radiologically visible, yet recurrence plausibly evolves from residual viable cells after surgery and chemotherapy, to micrometastatic expansion, molecularly detectable MRD, radiological recurrence and clinical recurrence. Molecular surveillance is translationally useful only if it converts part of this hidden interval into an action that can still change localization, resectability, trial access or patient burden.

Osteosarcoma-specific liquid biopsy data provide early support for this decision interval, although they do not yet define a standard management response. Pediatric solid tumor studies show that ctDNA assays based on structural variants and copy-number alterations may be more appropriate for childhood cancers than adult-style hotspot panels [21], [22], [23]. In osteosarcoma, early feasibility studies demonstrated detection of tumor-specific plasma alterations, while larger pediatric or osteosarcoma-containing cohorts linked ctDNA detectability or burden with tumor burden, response dynamics or inferior outcomes [21], [24], [32], [33], [34], [35]. Methylation-based ctDNA assays detected postoperative ctDNA in a subset of patients, and several positive results were associated with or preceded relapse [36]. Most directly, tumor-informed deep sequencing demonstrated that postoperative ctDNA status predicted event-free survival and, in a small subset of patients, preceded imaging-confirmed relapse or progression [25]. This finding provides preliminary osteosarcoma-specific evidence for a molecular-radiological lead-time interval, although the magnitude and consistency of this interval require validation in larger prospective cohorts. The surveillance implication is not simply that molecular relapse can be earlier than radiological relapse, but that some patients may enter a molecular-positive interval whose clinical value depends on whether disease can be localized and treated in time.

The translational interpretation is more important than the lead-time number itself. In recurrent osteosarcoma, outcome is repeatedly linked to disease burden, metastatic distribution and resectability. Late relapse, fewer nodules, unilateral pulmonary disease and achievement of second complete remission are favorable, whereas bilateral lung disease, multiple nodules, extrapulmonary metastases, local recurrence accompanying metastasis and progression during treatment identify patients less likely to undergo complete surgical clearance [6], [7], [9], [10], [11], [12], [13], [14]. Surveillance innovation should therefore aim to detect recurrence before the transition from solitary or oligometastatic disease to multifocal, less resectable dissemination. Molecular lead time becomes actionable lead time only if it triggers earlier anatomical confirmation, MDT review, metastasectomy assessment, local therapy planning, trial referral or a safe reduction in unnecessary surveillance burden.

## 4 Evidence layers for integrated surveillance

Integrated surveillance requires each biomarker to be interpreted as a layer of decision evidence rather than as an isolated test. In this section, liquid biopsy, anatomical imaging, functional MRI and radiomics are organized according to the surveillance function they can currently support: molecular timing, localization, local viability assessment and phenotypic risk enrichment.

### 4.1 Tumor-informed ctDNA as the strongest molecular timing signal

The decision question for liquid biopsy is not simply whether tumor-derived material can be detected in blood, but whether the assay can generate a dependable timing signal for postoperative risk management. ctDNA is the tumor-derived fraction of circulating cell-free DNA released from viable, apoptotic or necrotic tumor cells. Its surveillance appeal is repeated, minimally invasive sampling that may indicate residual tumor activity before a lesion becomes radiologically measurable. The technical challenge is that osteosarcoma lacks common recurrent point mutations and instead shows marked structural variation, copy-number alteration and inter- and intratumoral heterogeneity [21], [22], [23], [24]. Consequently, simple hotspot panels are unlikely to be sufficient. Tumor-agnostic strategies based on copy-number profiles, structural variation or methylation may be scalable, whereas tumor-informed strategies that first define patient-specific tumor alterations and then track them longitudinally may better fit the biology of a heterogeneous disease [21], [22], [25], [33].

The earliest osteosarcoma ctDNA studies should be read as design-enabling evidence rather than surveillance-changing evidence. Barris et al. used tumor-germline comparisons and a targeted panel to show that patient-specific somatic alterations could be detected in plasma from pediatric, adolescent and young adult patients, but the cohort was small and outcome correlation was limited [24]. Klega et al. and subsequent pediatric solid tumor studies showed that structural-variant and copy-number-based approaches could detect and quantify ctDNA in osteosarcoma and other childhood solid tumors, with serial levels sometimes reflecting treatment response or tumor burden [21], [33], [34], [35]. These studies justify plasma monitoring strategies and identify platform requirements, but they do not by themselves define postoperative MRD thresholds, sampling intervals, negative predictive value or management actions.

The strongest current osteosarcoma MRD evidence comes from tumor-informed longitudinal sequencing because it links postoperative molecular status with subsequent clinical course. Fu et al. performed whole-exome sequencing in 83 patients and confirmed substantial mutational heterogeneity, with most mutated genes being non-recurrent across patients. A customized MRD panel could be generated in 71 of 83 patients, and 59 patients with successful panel design and available serial blood samples were analyzed longitudinally [25]. Postoperative ctDNA positivity was observed in 13 patients and was associated with inferior event-free survival at clinically relevant time points, including 1–6 months after surgery, after adjuvant chemotherapy and beyond 6 months after surgery. In this cohort, ctDNA positivity, relapse timing and disease-free status represented different analytical outcomes rather than mutually exclusive patient categories. ctDNA positivity preceded imaging-confirmed relapse or progression in five patients by a mean of 92.6 days [25]. However, this lead-time estimate was derived from only five patients within a single-center cohort and should therefore be interpreted as preliminary, design-enabling evidence rather than as a stable estimate of the expected molecular-radiological lead time in osteosarcoma. This evidence supports the existence of a potential molecular-radiological timing gap in osteosarcoma, but its clinical meaning remains conditional: tumor-informed panel generation is not feasible in all patients, newly emerging subclones may not be represented in the original assay, low-shedding disease may yield false-negative results, and the optimal management of molecular-positive/imaging-negative states remains undefined.

### 4.2 Cell-free DNA methylation as a mutation-independent adjunct

Methylation-based cell-free DNA (cfDNA)/ctDNA detection addresses a different decision need: mutation-independent monitoring when trackable sequence variants are unavailable or insufficient. Lyskjær et al. developed osteosarcoma-specific methylation markers and tested methylation-specific droplet digital PCR (ddPCR) in plasma from 72 patients. ctDNA was detected in 69% of preoperative samples using a one-marker threshold and in 40% using a stricter two-marker threshold; postoperative ctDNA was detected in 5 of 17 available samples and, in four cases, was associated with or preceded relapse [36]. Both preoperative cfDNA levels and ctDNA detection independently correlated with overall survival [36]. For surveillance design, this evidence supports methylation as a candidate adjunct or fallback layer, but postoperative sample numbers were modest, positivity thresholds require validation, and assay performance may depend on plasma input and marker selection. Its current translational role is therefore to broaden molecular coverage in prospective workflows, not to provide a standalone relapse diagnosis.

### 4.3 Circulating tumor cells, extracellular vesicles and circulating microRNAs as exploratory signals

Other circulating biomarkers are best interpreted as complementary biological signals rather than near-term surveillance triggers. Circulating tumor cells (CTCs) provide a cellular measure of hematogenous dissemination and have been associated with stage, metastasis, chemotherapy response and disease-free survival in osteosarcoma [37], [38], [39]. Studies reporting mesenchymal CTC fractions, hexokinase 2 (HK2)-based CTC surveillance, insulin-like growth factor 2 mRNA-binding protein 3 (IMP3)-positive CTCs and hybrid epithelial/mesenchymal phenotypes suggest that circulating cells may capture metastatic behavior not fully represented by cfDNA [37], [38], [39]. However, osteosarcoma is a mesenchymal tumor, so epithelial-marker platforms are biologically mismatched; detection methods, enrichment strategies and cut-offs vary widely; and available studies remain small and platform-dependent. CTCs should therefore be positioned as exploratory indicators of dissemination, particularly for research into ctDNA-negative or low-shedding contexts, rather than as validated MRD triggers.

Extracellular vesicles (EVs) and circulating microRNAs (miRNAs) provide broader biological context, but current evidence mainly supports hypothesis generation. EVs can carry miRNAs, long non-coding RNAs (lncRNAs), proteins and DNA, participate in intercellular communication, and experimentally influence osteosarcoma proliferation, invasion, lung pre-metastatic niche formation, immune modulation and therapy resistance [40], [41]. Circulating miRNAs, including serum miR-25-3p and other signatures, have been associated with diagnosis, metastatic disease, tumor burden, survival or prognosis [42], [43], [44]. Yet candidate miRNAs differ across studies, cut-offs are inconsistent, and most data address diagnosis or baseline prognosis rather than serial postoperative MRD detection. These analytes may eventually refine multi-analyte risk models, but they should not distract from the stronger current evidence base for ctDNA and cfDNA methylation.

For clinical translation, liquid biopsy evidence should be organized by what it can validly decide. Analytically, ctDNA and cfDNA are the most mature circulating analytes in sarcoma, but assay performance varies by platform, tumor burden, histological subtype, pre-analytical workflow, sample volume, sequencing depth and positivity threshold [26], [27], [28], [45]. Osteosarcoma-specific biology favors tumor-informed sequencing, structural-variant/copy-number approaches and methylation strategies over hotspot panels, but each approach has trade-offs. Clinical validity is strongest where serial ctDNA positivity correlates with event-free survival, relapse or progression, particularly in the postoperative setting [25], [36]. Clinical utility is least established. A positive result should therefore trigger predefined reassessment, not automatic treatment escalation; a negative result should not justify imaging de-escalation until negative predictive value and safety have been prospectively tested. These liquid-biopsy decision roles are carried forward into the consolidated molecular-imaging readiness summary.

### 4.4 Chest CT and local MRI as the anatomical localization backbone

Imaging biomarkers answer a different decision question from liquid biopsy. Liquid biopsy may indicate when disease is biologically active; imaging determines where disease is and whether intervention is possible. Chest CT is central for pulmonary relapse, while local MRI remains essential for the primary tumor bed, reconstructive complications and local recurrence. This localization function is why current guidelines and consensus statements continue to place imaging at the center of follow-up [2], [15], [16], [17], [18]. In an integrated framework, imaging is not a competing technology but the anatomical confirmation layer required for biopsy, metastasectomy, radiotherapy, local surgical planning or trial eligibility.

This localization role should govern how molecular evidence is used. A molecular-positive/imaging-negative state may represent early relapse biology, but it may also reflect assay noise, transient low-level detectability or disease that is not yet localizable. It should therefore prompt repeat molecular testing and short-interval anatomical reassessment, not immediate systemic therapy. Conversely, an imaging-suspicious/molecular-negative finding should remain actionable because low-shedding pulmonary disease or anatomically confined local recurrence may not release sufficient ctDNA. Chest CT and local MRI should therefore remain the clinical backbone against which liquid biopsy and quantitative imaging are tested.

### 4.5 Diffusion-weighted imaging/apparent diffusion coefficient and dynamic contrast-enhanced MRI as local viability phenotypes

Functional MRI evidence should be used as local viability evidence, not as proof of systemic surveillance capability. Diffusion-weighted imaging (DWI) and apparent diffusion coefficient (ADC) mapping are the most widely studied functional MRI biomarkers in osteosarcoma. Highly cellular viable tumor tends to restrict water diffusion and show lower ADC values, whereas treatment-induced necrosis and extracellular expansion tend to increase ADC. Multiple studies have found that post-chemotherapy ADC values, ADC change or ADC ratios correlate with histological necrosis and may differentiate good from poor responders before resection [46], [47], [48], [49], [50]. A meta-analysis of 13 studies and 303 patients reported that ADC change and ADC ratio were significantly higher in good responders than poor responders, supporting DWI as a quantitative response biomarker [51]. More recent work using volumetric ADC or DWI-based radiomics suggests that spatial heterogeneity may improve response prediction beyond visual MRI assessment alone [46], [52]. These findings are surveillance-relevant because poor response and residual viability define a higher-risk context, but most DWI evidence still uses histological response rather than prospective relapse as the primary endpoint.

Dynamic contrast-enhanced MRI (DCE-MRI) provides a perfusion and vascularity phenotype that is closer to residual viability than to whole-body MRD. Viable osteosarcoma compartments enhance earlier and more intensely than necrotic, fibrotic or hemorrhagic tissue, allowing DCE-derived variables to act as surrogates of residual tumor activity. In a multicenter model-development and external-validation study, relative wash-in rate (rWIR) <2.3 identified poor radiological response and predicted histological response [53]. In a subsequent prognostic analysis from the same collaborative group, using a partly overlapping cohort of 82 patients, poor radiological response defined by rWIR <2.3 was associated with shorter event-free survival, with 2- and 5-year event-free survival rates of 55% and 50% compared with 85% and 75% for patients with rWIR ≥2.3; this association remained significant after adjustment for clinical covariates [54]. Although these studies provide complementary evidence supporting the prognostic relevance of rWIR, the shared patient population should be considered when interpreting the strength of evidence. A subsequent correction clarified the rWIR good/poor responder definition in the published tables without changing the authors' scientific conclusions. Prospective DCE-MRI and intravoxel incoherent motion DWI (IVIM-DWI) data further suggest that perfusion- and diffusion-related parameters can change early during chemotherapy and correlate with response [55]. The current surveillance role of DCE-MRI is therefore risk-contextual: it may help identify patients who warrant closer local assessment and integrated risk monitoring, but it cannot independently replace chest surveillance or detect systemic MRD.

### 4.6 Radiomics and radiogenomics as risk-enrichment tools

Radiomics converts standard images into quantitative descriptors of shape, margin, intensity, texture and spatial heterogeneity that may reflect tumor biology beyond conventional reporting [56], [57], [58]. Its appeal for risk-adapted surveillance is that osteosarcoma relapse risk reflects chemotherapy resistance, local invasiveness, metastatic potential and biological heterogeneity, not only visible tumor size. Radiogenomics extends this logic by linking imaging phenotypes with molecular profiles, potentially offering a non-invasive surrogate for tumor biology when tissue is incomplete or serial sampling is limited. For a surveillance review, however, radiomics should be framed primarily as a risk-enrichment tool until prospective evidence shows that acting on radiomics scores improves clinical outcomes.

Existing osteosarcoma radiomics studies therefore provide risk-enrichment evidence, not yet surveillance-intervention evidence. Pretreatment relapse-risk models have used CT or MRI to predict early recurrence, DWI-derived radiomics signatures have been associated with overall survival, positron emission tomography (PET)-derived metabolic phenotypes and multiparametric MRI radiomics have been used to predict metastatic risk, and CT/MRI radiomics models have been applied to neoadjuvant chemotherapy response [52], [59], [60], [61], [62], [63]. More recent multimodality and pediatric MRI-based studies have extended the field toward local recurrence, progressive disease, relapse, response and survival stratification [64], [65]. These data justify trial enrichment and hypothesis generation, but most models do not yet define an imaging-triggered surveillance action.

The translational limitation is that discrimination metrics alone do not define clinical utility. Many models are retrospective, derived from modest sample sizes, trained in single-center or regionally selected cohorts, and externally validated only in small datasets. Imaging sequences, scanner parameters, segmentation strategies, preprocessing, feature extraction and feature selection differ substantially across studies [59], [60], [64], [65], [66], [67], [68]. Area under the curve (AUC) alone is therefore insufficient. Calibration, decision-curve analysis, clinical impact, reproducibility, prospective validation, open model reporting and integration into radiology workflow are essential. Transparent feature definitions and reproducible extraction pipelines such as PyRadiomics are necessary but not sufficient for clinical adoption [56]. Radiomics should currently be used to generate and test risk hypotheses, not to dictate surveillance intensity outside research protocols. Table 1 consolidates these molecular and imaging decision-readiness layers.

**Table 1:** Decision readiness of molecular and imaging biomarkers for osteosarcoma relapse surveillance.

Surveillance layer	Biomarker or modality	Best-supported signal	Current decision role and action boundary	References
Molecular timing	Tumor-informed ctDNA	Postoperative ctDNA positivity is the strongest osteosarcoma-specific MRD signal reported to date; In Fu et al., ctDNA preceded imaging-confirmed relapse or progression in a small subset of patients, providing preliminary evidence of a molecular-radiological lead-time interval.	Research-ready molecular timing layer. A positive or rising result may trigger repeat blood testing, short-interval chest CT/local MRI, MDT review and trial screening, but should not trigger treatment escalation without anatomical or protocol-defined evidence.	[21], [22], [23], [24], [25] [32], [33], [34], [35]
Molecular adjunct	cfDNA/ctDNA methylation	Mutation-independent ctDNA detection has been reported in osteosarcoma, including postoperative positivity associated with or preceding relapse in a small subset.	Adjunct or fallback molecular layer when sequence-based tracking is unavailable or incomplete. Positive results require confirmation and anatomical reassessment; they should not be used as standalone relapse diagnoses.	[20], [36]
Exploratory dissemination signal	CTCs	CTC burden, marker-defined CTCs and epithelial/mesenchymal phenotypes have been associated with metastatic features, treatment response or DFS in small, platform-dependent cohorts.	Exploratory research layer, especially for low-shedding or ctDNA-negative contexts. CTC counts or phenotypes are not validated MRD triggers and should not guide routine surveillance decisions.	[37], [38], [39]
Exploratory multi-analyte biology	Extracellular vesicles and circulating miRNAs	EV and miRNA studies suggest links with metastatic biology, therapy resistance, tumor burden or prognosis, but serial postoperative surveillance evidence is limited.	Discovery and multi-analyte model-development layer. EV or miRNA signals should not be used to diagnose relapse, modify treatment or alter imaging frequency outside research.	[40], [41], [42], [43], [44]
Anatomical localization	Chest CT and local MRI	Chest imaging and local-site MRI remain the clinical backbone for detecting pulmonary relapse, local recurrence and lesions requiring biopsy, metastasectomy, radiotherapy or surgical planning.	Mandatory localization layer. Suspicious imaging remains actionable even when ctDNA is negative; MRD-positive/imaging-negative states should trigger structured reassessment, not treatment without localizable disease.	[2], [15], [16], [17], [18] [29], [30], [31]
Local viability phenotype	DWI/ADC	ADC values, ADC change and ADC ratios correlate with histological necrosis and chemotherapy response, but most evidence is response-based rather than relapse-surveillance based.	Candidate local viability and risk-context layer. It may support closer assessment in research protocols, but is not a standalone systemic MRD marker or independent relapse-surveillance trigger.	[46], [47], [48], [49], [50] [51], [52]
Perfusion phenotype	DCE-MRI/rWIR	rWIR has been linked to histological response and EFS in multicenter osteosarcoma studies, although action thresholds remain unvalidated for surveillance.	Candidate perfusion-based risk layer. It may enrich integrated monitoring strategies, but should not replace chest surveillance or guide treatment escalation without prospective validation.	[53], [54], [55]
Quantitative risk enrichment	Radiomics/radiogenomics	CT, MRI, DWI, PET and multimodality radiomics models have been reported for relapse, metastasis, response and survival stratification, but most remain retrospective and variably validated.	Trial-enrichment and hypothesis-generating layer. Radiomics scores should not independently alter follow-up intensity or treatment until calibration, external validation, decision-curve analysis and workflow feasibility are shown.	[59], [60], [61], [62], [63] [64], [65], [69], [70]
Implementation standards	Liquid-biopsy and radiomics methodology	Clinical deployment requires analytical validity, clinical validity, clinical utility, transparent pre-analytics, reproducible imaging pipelines, external validation and reporting of indeterminate results.	Methodological gatekeeper. Biomarkers should be embedded in prospective protocols with prespecified action and non-action thresholds, not interpreted ad hoc in routine surveillance.	[20], [27], [28], [45], [56] [57], [58], [66], [67], [68] [71], [72], [73], [74]

Abbreviations: ADC, apparent diffusion coefficient; cfDNA, cell-free DNA; CT, computed tomography; CTC, circulating tumor cell; ctDNA, circulating tumor DNA; DCE-MRI, dynamic contrast-enhanced magnetic resonance imaging; DFS, disease-free survival; DWI, diffusion-weighted imaging; EFS, event-free survival; EV, extracellular vesicle; MDT, multidisciplinary team; miRNA, microRNA; MRD, minimal residual disease; MRI, magnetic resonance imaging; PET, positron emission tomography; rWIR, relative wash-in rate.

## 5 Action-linked surveillance framework

### 5.1 Why single-modality monitoring is insufficient

No single evidence stream captures all clinically relevant dimensions of osteosarcoma relapse. Conventional imaging localizes pulmonary metastases, local recurrence and resectable lesions, but it usually becomes positive only after disease reaches a measurable anatomical threshold. The lead-time signal reported by Fu et al. provides preliminary evidence that molecular detection may precede radiological confirmation in selected patients, supporting prospective evaluation of biomarker-triggered reassessment rather than defining a fixed surveillance interval [25]. However, ctDNA can be falsely negative in low-shedding disease, may miss subclones not represented in the original tumor-informed panel and does not localize disease for curative intervention. Functional MRI and radiomics add local viability and quantitative risk information, but most evidence derives from response prediction or retrospective risk modeling rather than prospective surveillance trials [51], [54], [59], [60], [66], [67]. The logical solution is integration rather than substitution: molecular timing to detect risk, anatomical imaging to localize disease, and imaging-derived phenotypes to stratify who may benefit from closer follow-up.

### 5.2 Risk inputs and dynamic updating

A practical surveillance workflow can combine three input layers, each supported by a different type of evidence. The first is established clinicopathological risk: metastatic disease at diagnosis, axial or proximal tumor site, large tumor volume, elevated alkaline phosphatase or lactate dehydrogenase, surgical margin status and poor histological response to preoperative chemotherapy [2], [3]. The second is molecular risk: postoperative ctDNA or cfDNA methylation positivity, persistent positivity after adjuvant chemotherapy, dynamic conversion from negative to positive, or rising quantitative burden [25], [36]. The third is imaging-derived risk: suspicious chest CT or local MRI findings, poor functional MRI response such as limited ADC increase or low rWIR, and candidate high-risk radiomics signatures [51], [54], [59], [60]. The purpose is not to replace clinical judgment with a score, but to update risk as new evidence appears during treatment and follow-up.

Dynamic prediction is appropriate because osteosarcoma risk changes over time and because surveillance decisions are sequential. A retrospective analysis of EURAMOS-1 data showed that updated follow-up information, including local recurrence and new metastatic disease, improved patient-specific survival estimation compared with baseline-only models [75]. A next-generation surveillance system should apply the same principle prospectively. Baseline clinicopathological factors define initial risk, but postoperative MRD status, biomarker conversion, chest CT findings, local MRI features, functional MRI response and radiomics signatures should update that risk as follow-up progresses. The intended output is not a static risk label; it is a longitudinal decision model that indicates when to maintain standard follow-up, bring imaging forward, repeat molecular testing, convene MDT review or refer to a clinical trial.

### 5.3 Molecular-positive/imaging-negative pathway

The most important translational use of integrated surveillance may be the management of concordant and discordant results, because this is where biomarker evidence becomes clinical decision pressure. A molecular-positive/imaging-negative state is the signature scenario for MRD surveillance. It should be interpreted as a credible risk signal, especially if repeated or rising, but not as a relapse diagnosis by itself. The appropriate research action is repeat blood sampling, earlier chest CT, targeted local MRI and MDT review, with biopsy or local therapy only if disease becomes anatomically localizable. This protects against both delayed confirmation and unsupported systemic treatment.

### 5.4 Imaging-positive/molecular-negative pathway

An imaging-suspicious/molecular-negative state remains actionable because a negative liquid biopsy result is not equivalent to absence of disease. Low-shedding lung nodules, anatomically confined local recurrence, low plasma input or subclonal mismatch may all produce false-negative molecular results. The appropriate action is radiology review, short-interval imaging, biopsy when feasible and continued clinical assessment rather than reassurance based on MRD negativity.

### 5.5 Concordant high-risk and concordant low-risk pathways

A concordant molecular-negative/imaging-negative state is the only plausible de-escalation scenario, but even here de-escalation should remain a trial endpoint until negative predictive value, patient safety and recurrence patterns are prospectively established.

A concordant molecular-positive/imaging-positive state identifies the highest-priority pathway for MDT action because timing and localization evidence point in the same direction. The clinical question shifts from whether relapse exists to whether complete resection, metastasectomy, radiotherapy, systemic therapy, molecular re-profiling or trial referral is feasible. In this setting, biomarker data may support urgency, risk communication and trial selection, but the action still depends on anatomical extent and multidisciplinary judgment. This four-state structure is the practical bridge between biomarker evidence and clinical workflow.

### 5.6 Proposed risk categories and candidate actions

Low-, intermediate- and high-risk categories should be treated as trial hypotheses rather than clinical instructions. Low-risk patients might include those with localized extremity disease, complete resection with clear margins, good histological response, negative postoperative MRD and low-risk imaging features. Standard guideline-based follow-up would remain appropriate, with biomarker testing used as confirmatory research monitoring rather than a basis for immediate de-escalation. Intermediate-risk patients might have one adverse clinicopathological factor, equivocal imaging risk, poor radiological response or a single abnormal biomarker result without anatomical confirmation. Reasonable research actions include repeat liquid biopsy, short-interval chest CT or local MRI, radiology review and MDT discussion. High-risk patients might include those with metastatic disease at diagnosis, poor histological response, close or positive margins, axial or proximal tumors, persistent postoperative ctDNA positivity, candidate high-risk radiomics features or poor functional MRI response. Appropriate endpoints to test in this group include intensified multimodal surveillance, earlier anatomical confirmation, metastasectomy assessment, local therapy planning and clinical trial referral.

Importantly, no risk category should be used to reduce imaging intensity, escalate treatment or reassure patients outside a predefined protocol. The clinical-translational value of the framework is not that it creates three labels, but that it forces every label to be linked to an auditable action and an endpoint. Fig. 1 summarizes this evidence-calibrated workflow as a research model rather than a clinical guideline.

**Fig. 1:**
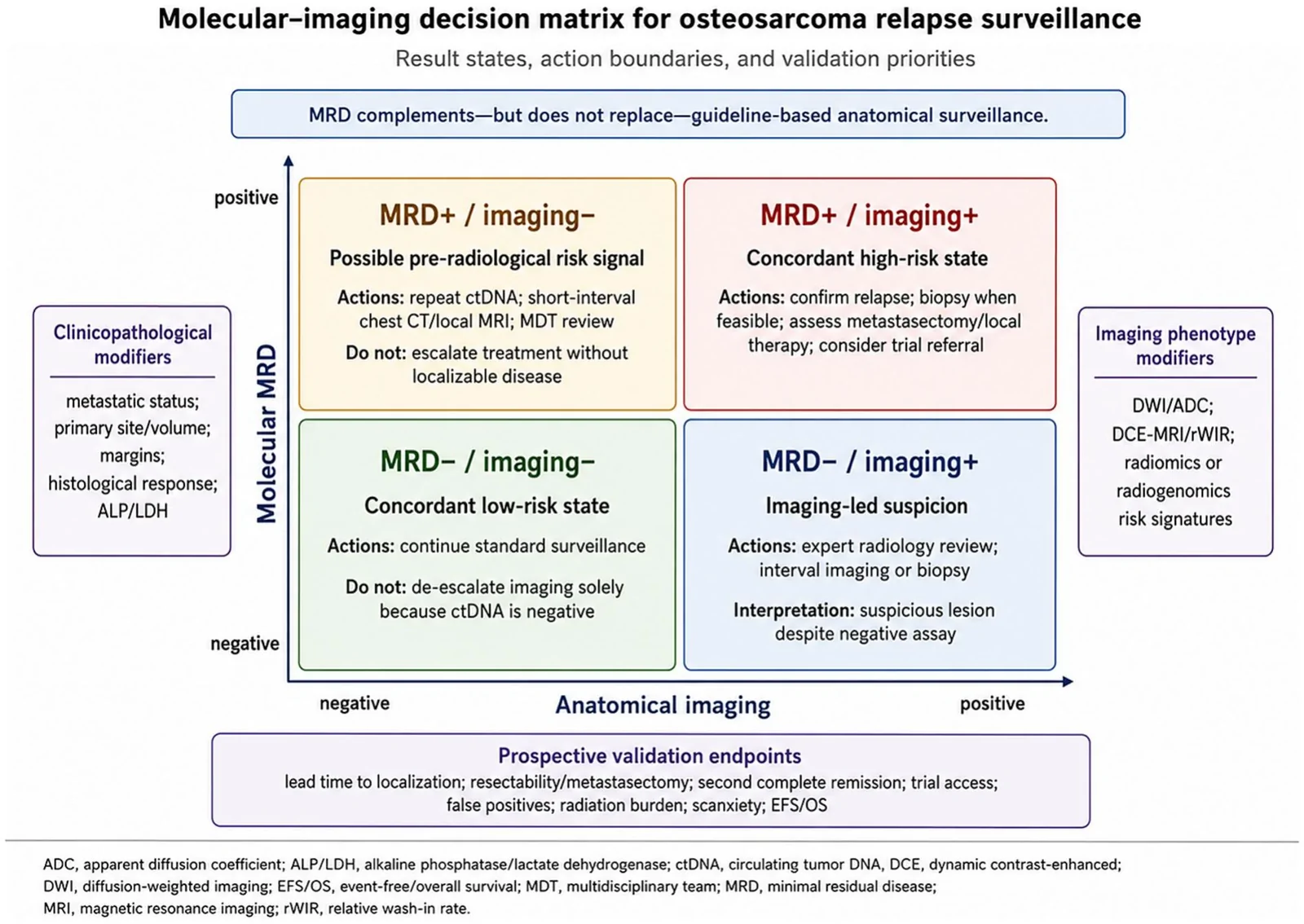
Molecular-imaging decision matrix for osteosarcoma relapse surveillance.

### 5.7 Action thresholds, treatment thresholds and non-actions

For clinical translation, the framework should distinguish action thresholds from treatment thresholds. A single molecular-positive result may be enough to trigger confirmation, but not enough to define relapse or justify systemic therapy. A persistent or rising ctDNA signal may justify earlier chest CT, targeted local MRI and MDT discussion, but treatment escalation still requires anatomical, pathological or trial-defined evidence. Similarly, a high-risk radiomics score may justify closer review or trial enrichment, but not independent alteration of standard follow-up. This distinction is essential because the cited literature supports prognostic association and candidate lead time more strongly than treatment benefit.

Candidate actions should be deliberately modest at the validation stage. Bringing chest CT forward, adding targeted local MRI, repeating liquid biopsy, reviewing radiomics outputs, discussing the case in an MDT meeting, referring for clinical trial screening, or planning biopsy when disease becomes localizable are plausible actions. Candidate non-actions are equally important: do not start systemic therapy solely for molecular positivity without localizable disease; do not ignore suspicious imaging because ctDNA is negative; do not reduce imaging simply because MRD is negative; and do not apply adult carcinoma MRD thresholds directly to osteosarcoma. These non-actions protect patients from overinterpretation while preserving the possibility of earlier salvage.

A practical protocol should therefore contain explicit stop-go rules: how soon a positive test must be repeated, what constitutes molecular persistence or rise, which imaging modality is triggered, how equivocal nodules or surgical-bed findings are handled, when biopsy is required, when MDT review is mandatory, and what endpoint each action is intended to improve. Without such rules, integrated surveillance risks becoming more intensive but not more useful.

## 6 Standardization, validation and implementation

### 6.1 Standardizing linked liquid biopsy and imaging workflows

The main barrier to translation is not the absence of promising biomarkers, but the absence of standardized workflows that make evidence comparable, interpretable and actionable across institutions. Liquid biopsy implementation requires harmonization of blood collection time points, tube type, plasma processing delay, storage conditions, input volume, DNA/RNA extraction, assay platform, analytical sensitivity, positivity threshold, failure rules and reporting format [20], [27], [28], [45]. In osteosarcoma, blood sampling should be synchronized with clinically meaningful milestones: diagnosis, during neoadjuvant chemotherapy, preoperatively, early postoperatively, during adjuvant chemotherapy and at prespecified surveillance visits. Opportunistic sampling will not allow reliable estimation of lead time, negative predictive value or biomarker kinetics, and therefore cannot support decision-grade surveillance evidence.

Imaging standardization is equally important because quantitative imaging evidence is only useful if an observed change reflects tumor biology rather than acquisition variation. DWI, DCE-MRI and radiomics require reproducible CT and MRI protocols, scanner documentation, field strength, slice thickness, contrast timing, b-values, DCE temporal resolution, reconstruction parameters, segmentation methods, feature extraction and calibration. Radiomics studies should report preprocessing, feature selection, model training, data partitioning, external validation, clinical utility analysis and code or feature definitions whenever feasible [56], [66], [67], [68], [71], [72], [73]. Harmonization does not mean every center must use identical equipment; it means that technical variation must be measured, minimized or modeled before biomarker changes are interpreted as evidence for a surveillance decision.

### 6.2 Designing prospective biomarker-guided surveillance studies

Future studies must move beyond asking whether biomarkers predict relapse and ask whether biomarker-guided surveillance improves management. The supporting evidence should be translated into study-design requirements rather than treated as a clinical guideline. The most informative first step for osteosarcoma would be a prospective, multicenter, longitudinal cohort embedded within routine follow-up. Patients should be enrolled at diagnosis or at the completion of curative-intent therapy, tumor tissue should be collected for tumor-informed MRD when planned, and serial blood sampling should be synchronized with standardized chest CT and local MRI intervals. Molecular conversion, imaging changes and clinical relapse should then be mapped on the same timeline so that each biomarker signal can be evaluated as a decision trigger rather than as an isolated prognostic observation. External solid-tumor experience supports this logic but should not be overgeneralized: ctDNA studies in localized lung cancer showed that molecular relapse may precede radiological progression, whereas the randomized DYNAMIC trial in stage II colon cancer demonstrated that ctDNA-guided management can be tested as an interventional strategy with predefined actions and clinical endpoints [76], [77]. Osteosarcoma needs the same type of action-linked evidence, not simple transfer of thresholds from other tumors.

### 6.3 Endpoints beyond earlier detection: Resectability, second remission, trial access and patient burden

Predefined endpoints should distinguish earlier detection from improved care. They should include radiological relapse, event-free survival, overall survival, time from biomarker positivity to imaging relapse, proportion of biomarker-positive episodes that become anatomically localizable, resectability at recurrence, metastasectomy rate, achievement of second complete remission, local therapy planning, treatment modification, trial enrollment, false-positive investigations, radiation and visit burden, patient anxiety and quality of life. For ctDNA, protocols should prespecify positivity thresholds, panel failure rules, minimum plasma input, turnaround time and management of molecular-positive/imaging-negative results [19], [20], [25]. For methylation, CTCs, extracellular vesicles and miRNAs, assay normalization and cut-offs must be reproducible before clinical interpretation. For imaging biomarkers, thresholds such as rWIR <2.3 or radiomics risk scores require prospective testing against predefined relapse and survival endpoints rather than retrospective optimization [54], [59], [60]. Diagnostic-accuracy reporting standards such as the Standards for Reporting Diagnostic Accuracy Studies (STARD) provide a useful reminder that sensitivity, specificity, timing, reference standards and indeterminate results should be reported transparently before a surveillance test is treated as decision-grade evidence [74].

Discordant-result pathways should also be prespecified because they determine whether evidence becomes responsible action or uncontrolled escalation. A molecular-positive/imaging-negative state should trigger repeat blood sampling, short-interval chest CT, local MRI review and MDT discussion rather than immediate systemic therapy without localizable disease. An imaging-suspicious/molecular-negative state should remain actionable because ctDNA shedding can be low and local recurrence may be anatomically confined. A concordant high-risk state may justify trial referral or intensified surveillance, whereas a concordant low-risk state should support de-escalation only after safety is proven. This evidence-to-action mapping would make future studies auditable and would separate biomarker enthusiasm from clinically responsible implementation. In a later phase, the field could move from observational validation to response-adaptive or randomized utility trials, but only after feasibility, assay turnaround, false-positive rates and patient burden have been quantified in osteosarcoma-specific cohorts.

### 6.4 MDT implementation, patient communication and equity

MRD-guided surveillance should not simply add blood tests to fixed imaging schedules; it should personalize follow-up intensity only when personalization is shown to be safe and useful. Persistently negative MRD, low-risk imaging features and favorable clinicopathological factors could be tested as criteria for surveillance de-escalation, including lower imaging frequency, reduced radiation exposure, fewer hospital visits, less sedation burden and lower scan-related anxiety. Conversely, persistent or rising ctDNA, high-risk cfDNA methylation, suspicious CTC dynamics, adverse radiomics signatures or poor functional MRI response could justify earlier imaging and MDT review within a predefined research protocol [25], [26], [54]. Patient burden must be measured rather than assumed. A systematic review of surveillance imaging after treatment for childhood extracranial solid tumors found insufficient evidence that routine imaging improves survival in most diseases and highlighted radiation exposure, scan volume, cost-effectiveness uncertainty and the lack of psychological distress data as major gaps [78]. A broader systematic review of postoperative cancer surveillance reported that scanxiety is common, often concentrated around the scan-to-results interval, and not reliably reduced by simply modifying surveillance schedules [79]. These data support inclusion of patient-reported anxiety, waiting time, false-positive investigations, cumulative imaging exposure and family burden as endpoints in osteosarcoma surveillance studies.

Implementation also determines whether promising evidence can become a bone oncology workflow. Tumor-informed assays require tumor tissue availability, sequencing capacity, bioinformatics support and turnaround times compatible with clinical milestones. However, real-world implementation is constrained by assay feasibility. In the Fu et al. study, customized MRD panels could not be generated in 12 of 83 patients, indicating that tumor-informed monitoring may not be universally applicable because of insufficient sequencing targets, technical limitations or other assay-related factors [25]. For patients without an available tumor-informed assay, standard imaging-based surveillance remains essential, and alternative molecular approaches should currently be considered investigational rather than assumed replacements for ctDNA monitoring. In pediatric and adolescent populations, longitudinal liquid biopsy strategies also require consideration of patient-specific factors, including blood volume limitations, venous access, sampling frequency and cumulative burden associated with repeated blood collection [26].

Imaging biomarkers require radiology workflows that generate quantitative results reproducibly without excessive manual burden. Multidisciplinary interpretation is essential because a molecular pathologist, radiologist, orthopedic oncologist, medical oncologist and survivorship team may assign different meaning to the same result. Furthermore, cost, reimbursement, access to specialized sequencing platforms, turnaround time and resource availability may limit implementation outside tertiary referral centers. Prospective studies should therefore evaluate not only analytical performance and prognostic association, but also workflow feasibility, assay failure rates, reporting timelines, patient burden and equity of access before biomarker-guided surveillance can be incorporated into routine osteosarcoma care.

## 7 Conclusions

Conventional imaging-based follow-up remains essential in osteosarcoma because relapse must be anatomically confirmed before curative salvage can be attempted. However, imaging alone does not fully capture the earlier biological phases of recurrence in which residual viable cells, micrometastatic disease and molecular relapse may precede radiological visibility. This review argues that the next step is not a single replacement biomarker, but an action-linked surveillance model that connects molecular timing, anatomical localization, imaging-derived phenotype, discordant-result management and predefined clinical action. Tumor-informed ctDNA currently provides the strongest molecular MRD evidence, while cfDNA methylation, CTCs, extracellular vesicles and circulating miRNAs remain complementary but less mature signals. Functional MRI and radiomics contribute local viability and candidate risk-stratification information, but most imaging biomarker evidence still derives from response, prognosis or retrospective modeling rather than validated surveillance intervention. Clinical translation will require harmonized assays, reproducible imaging protocols, explicit action and non-action thresholds, prespecified discordant-result pathways, multicenter longitudinal validation and endpoints that show improved resectability, trial access, patient-centered burden or survival. Integrated biomarker-guided surveillance may become a clinically useful bone oncology workflow only if molecular or imaging lead time can be converted into actionable benefit rather than earlier documentation of relapse alone.

## CRediT authorship contribution statement

**Guoli Huang:** Writing – review & editing, Writing – original draft, Methodology, Investigation, Data curation, Conceptualization. **Yang Zhou:** Writing – review & editing, Investigation, Data curation. **Hao Wang:** Writing – review & editing, Investigation, Data curation. **Xiucai Ma:** Writing – review & editing, Supervision, Project administration, Methodology, Funding acquisition, Conceptualization.

## Funding

Funding: This work was supported by the Young Science and Technology Talent Innovation Project, 10.13039/501100007764Science and Technology Bureau of Lanzhou [grant number 2023-4-58]; the Gansu Provincial Health Commission Project [grant number GSWSKY2021–013]; and the 10.13039/501100004775Natural Science Foundation of Gansu Province [grant number 24JRRA1059].

## Declaration of competing interest

The authors declare that they have no known competing financial interests or personal relationships that could have appeared to influence the work reported in this paper.
